# Root symbiotic fungi improve nitrogen transfer and morpho-physiological performance in *Chenopodium quinoa*

**DOI:** 10.3389/fpls.2024.1386234

**Published:** 2024-08-26

**Authors:** Shirley Alquichire-Rojas, Elizabeth Escobar, Luisa Bascuñán-Godoy, Marcia González-Teuber

**Affiliations:** ^1^ Facultad de Ciencias, Universidad Católica de la Santísima Concepción, Concepción, Chile; ^2^ Departamento de Botánica, Facultad de Ciencias Naturales y Oceanográficas, Universidad de Concepción, Concepción, Chile; ^3^ Facultad de Ciencias Biológicas, Pontificia Universidad Católica de Chile, Santiago, Chile

**Keywords:** entomopathogenic fungi, nitrogen transfer, photosynthesis, carbon allocation, plant growth, symbiosis, quinoa

## Abstract

Root-associated fungal endophytes may facilitate nitrogen (N) absorption in plants, leading to benefits in photosynthesis and growth. Here, we investigated whether endophytic insect pathogenic fungi (EIPF) are capable of transferring soil N to the crop species *Chenopodium quinoa*. We evaluated nutrient uptake, carbon allocation, and morpho-physiological performance in *C. quinoa* in symbiosis with two different EIPF (*Beauveria* and *Metarhizium*) under contrasting soil N supply. A controlled experiment was conducted using two plant groups: (1) plants subjected to low N level (5 mM urea) and (2) plants subjected to high N level (15 mM urea). Plants from each group were then inoculated with different EIPF strains, either *Beauveria* (EIPF1+), *Metarhizium* (EIPF2+) or without fungus (EIPF-). Differences in N and C content, amino acids, proteins, soluble sugars, starch, glutamine synthetase, glutamate dehydrogenase, and physiological (photosynthesis, stomatal conductance, transpiration), and morphological performance between plant groups under each treatment were examined. We found that both *Beauveria* and *Metarhizium* translocated N from the soil to the roots of *C. quinoa*, with positive effects on photosynthesis and plant growth. These effects, however, were differentially affected by fungal strain as well as by N level. Additionally, an improvement in root C and sugar content was observed in presence of EIPF, suggesting translocation of carbohydrates from leaves to roots. Whereas both strains were equally effective in N transfer to roots, *Beauveria* seemed to exert less demand in *C. quinoa* for photosynthesis-derived carbohydrates compared to *Metarhizium*. Our study revealed positive effects of EIPF on N transfer and morpho-physiological performance in crops, highlighting the potential of these fungi as an alternative to chemical fertilizers in agriculture systems.

## 1 Introduction

Nitrogen (N) is among the most limiting nutrients for plant growth (Stewart et al., 2005). N has a principal role in the synthesis of nucleic acids, amino acids, and proteins, and is a major contributor to photosynthetic proteins and pigments in plants (Miller and Cramer, 2005; Svennerstam et al., 2008; Guo et al., 2020; Muratore et al., 2021; Llebrés et al., 2022). N is taken up by roots and transformed into organic molecules in both roots and leaves by different enzymes, including glutamate dehydrogenase and glutamine synthetase, which incorporate NH_4_^+^ into amino acids (de la Peña et al., 2019). About 60% of N in plants is stored in forms such as Rubisco, which is the limiting enzyme in the carbon fixation process. In order to overcome N limitation, plants establish symbiotic relationships with a range of microorganisms, such as rhizobial bacteria and soil fungi, including mycorrhiza and endophytic fungi (Udvardi and Poole, 2013; Bücking and Kafle, 2015; Wang et al., 2017). Besides nitrogen fixing bacteria such as rhizobia, which are responsible for nodulation and N_2_-fixation, fungi may play an important role in N transfer from the soil to roots. Thus, root-associated microorganisms facilitate the absorption of N, which may lead to increased photosynthetic efficiency and enhanced plant growth and productivity (Chen et al., 2020; Das et al., 2022).

Root-colonizing fungi, including arbuscular mycorrhiza, ectomycorrhizal as well as endophytic fungi, form symbiosis with lateral roots of plants and create an extraradical mycelium (ERM), which penetrates the intercellular spaces between the cortical root cells, forming the intraradical mycelium (IRM) (Smith and Read, 2008). According to current knowledge, NO_3_- and NH_4_+ are the primary N sources taken up by fungi. N is converted into arginine in the ERM, which is the main form in which N is transported from the ERM to the IRM. Once in the IRM, arginine is metabolized into ammonium and subsequently released to the symbiotic interface, where it is acquired and assimilated by plants (Behie and Bidochka, 2014a; Wang et al., 2017; Rui et al., 2022). While the mechanisms of N transport are relatively well known for mycorrhiza fungi, the ability of fungal endophytes to transfer N to the roots is a relatively recent finding. Since fungal endophytes are ubiquitous in soils and able to colonize a wide range of plants (from monocots to dicots) (Behie and Bidochka, 2014b), their N transfer capabilities may potentially be applied in agricultural systems as a means to increase productivity in crop species.

Numerous fungal endophyte strains increase nitrogen uptake efficiency in plants (Usuki and Narisawa, 2007; Behie and Bidochka, 2014b; González-Teuber et al., 2019). For example, the dark septate endophyte *Heterconium chaetospira* is able to transfer N obtained from decomposed soil organic material to the roots of *Brassica campestris* (Usuki and Narisawa, 2007). Additionally, endophytic, insect pathogenic fungi (EIPF) such as the genera *Metarhizium* and *Beauveria*, which colonize plant roots, have been shown to translocate soil nitrogen to different host plants (Behie et al., 2012; Behie and Bidochka, 2014a, b). *Beauveria* and *Metarhizium* infect soil-borne insects and have the ability to establish associations with host roots and transfer insect-derived nitrogen, which has been found to increase plant performance on the whole (Behie et al., 2012; Behie and Bidochka, 2014a; González-Pérez et al., 2022). Behie et al. (2012) demonstrated that, in association with *Metarhizium*, haricot bean and switchgrass derive approximately 30% of their N content from soil insects. Interestingly, similarly to mycorrhiza, N transfer to plant roots in this process occurs in exchange for photosynthetically fixed carbon (Kiers et al., 2011; Wang et al., 2017; Balestrini et al., 2020). In a labeling study using CO_2_ isotopes, Behie et al. (2015) showed (^13^CO_2_) that atmospheric CO_2_ was incorporated into plant carbohydrates, and subsequently translocated to *Metarhizium*-specific carbohydrates. This nutrient exchange of both partners appears necessary to maintain the plant-fungus symbiosis; nevertheless, how this exchange varies depending on the soil N level has been little explored (Barelli et al., 2019).

*Chenopodium quinoa* is a pseudo-cereal crop of the Chenopodiaceae family native to the Andean region of South America. Quinoa is an important crop species due to its high protein content and its resilience to stressful conditions (Bascuñán-Godoy et al., 2016; Lutz and Bascuñán-Godoy, 2017). Previous studies have shown that *C. quinoa* is able to establish symbiotic associations with numerous root endophytic fungi (González-Teuber et al., 2017), which benefit quinoa by improving plant morphological and physiological responses to abiotic stresses such as drought and salinity (González-Teuber et al., 2018; 2022). The role, however, of EIPF on the morpho-physiological performance of *C. quinoa* has not been addressed. Since EIPF genera *Beauveria* and *Metarhizium* are ubiquitous soil fungi able to transfer N from soil into roots (Behie et al., 2012), they have the potential to be applied in crop species as a means to increase their productivity. Here, we explored the question of whether EIPF are able to transfer N to *C. quinoa* from the soil without the need to infect insects. To do this, we evaluated nutrient uptake, carbon allocation, and morpho-physiological performance in *C. quinoa* in symbiosis with two different EIPF (*Beauveria* and *Metarhizium*) under contrasting soil N supply. We also discuss potential plant-fungus nutrient exchanges linked to soil N level.

## 2 Materials and methods

### 2.1 Study system

The Quinoa lowland genotype UdeC9 (latitude 35.73° S; longitude 72.53° W) was used for this study because it is highly susceptible to low nitrogen availability (Bascuñán-Godoy et al., 2018). UdeC9 seeds were provided by the National Seed Bank collection at Vicuña, Chile (INIA-Intihuasi). EIPF *Beauveria* were obtained from soils under vine crops in Viña Casanueva (Maule), Chile (36° 42´ 36´´ S; 72° 20´ 59´´ W) and *Metarhizium* in Viña Santa Rita (Alto Jahuel), Chile (33° 43´ 12´´ S; 70° 40´ 12´´ W). Specimens were isolated from soil samples using the *Tenebrio molitor* larval baiting technique (Meyling, 2007). One strain each of *Beauveria bassiana* and *Metarhizium* were selected for inoculation experiments. Identified morphologically, *Beauveria* showed hyaline and subglobose conidia, whereas *Metarhizium* showed cylindrical conidia with olive-green coloration, which is characteristic of the species (Aguilera-Sammaritano et al., 2021; Wang et al., 2020a). Both fungal genera were only identified through microscopical analysis. Nevertheless, DNA amplification with specific primers designed for both *Beauveria* and *Metarhizium* genera (see below) helped us to validate our original taxonomical identification. Both EIPF were grown in potato dextrose agar (PDA) for 15 days at 25°C. Fungal spores were then collected by repeatedly flooding the agar plates with sterile distilled water plus Tween 80 (0.01% v/v) and rubbing the surface with a sterile scraper. The samples were transferred to sterile bottles for storage. The spore concentration was adjusted to 1×10^7^ spores mL^-1^ by counting spores using a Neubauer chamber cell counting (HBG), and then used to inoculate the substrate directly by drenching.

### 2.2 Experimental design

The experiment was performed in a completed randomized design with a total of six treatments each containing 27–30 experimental units. The plants were divided into two groups: the first was fertilized with a single dose of a low-level N-urea solution (5 mM) while the second was fertilized with a high-level N-urea solution (15 mM). Both doses have previously been determined in *C. quinoa* through biomass curves under the supply of different amounts of N (Bascuñán-Godoy et al., 2018; Pinto-Irish et al., 2020; Jerez et al., 2023). After 15 days of vegetative growth, plants from each N level treatment were separated into three groups: (1) non-inoculated plants (EIPF-), (2) plants inoculated with *Beauveria* (EIPF1+), and (3) plants inoculated with *Metarhizium* (EIPF2+).

### 2.3 Plant growth conditions

*Chenopodium quinoa* seeds were surface-sterilized in 0.5% sodium hypochlorite for 3 minutes, triple-rinsed in sterile distilled water and then germinated on sterilized paper in petri dishes over a period of 24 hours in darkness before being sown on sterilized sand. Germinated seeds were transplanted individually in 0.52 L pots with sterile coarse sand that had previously been autoclaved at 120°C for 40 minutes. Plants were supplied once on planting day with MS solution nutrient medium, described by Murashige and Skoog (1962), consisting of 0.30 mM MgSO_4_.7H_2_O, 0.22 mM CaCl_2_, 0.62 mM KH_2_PO_4_, 12.7 mM KCl, 0.05 μM KI, 1.00 μM H_3_BO_3_, 1.32 μM MnSO_4_.4H_2_O, 0.30 μM ZnSO_4_.7H_2_ O, 0.01 μM Na_2_MoO_4_.2H_2_O, 0.001 μM CuSO_4_.5H_2_O, 0.001 μM CoCl_2_.6H_2_O, 0.51 μM Na_2_-EDTA, 0.50 μM FeSO_4_.7H_2_O. N-urea varied according to treatment. pH was set at 5.8. All plants in all treatments were then watered with additional distilled water as required. To avoid effects of microclimatic variations due to pot position, plants were randomly rearranged once a week. Plants were grown in a chamber at 20–25°C with a light/dark cycle of 12 h:12 h at a relative humidity of ~70% for 33 days. The photosynthetically active photon flux density (PPFD) ranged from 700 to 800 µmol m^-2^ s^-1^. After 15 days of vegetative growth, plants were watered with 30 mL spore solution of either *Beauveria* or *Metarhizium*. Non-inoculated plants at each N level were irrigated with sterile spore-free water. Morphological and physiological traits were measured after 15 days of applied treatments, including above- and below-ground biomass, photosynthesis, stomatal conductance, and transpiration. Additionally, leaf and root material from remaining plants were collected, and immediately frozen in liquid nitrogen and stored at -80°C for further measurements of biochemical parameters. Root frozen material was also used for DNA extraction and further fungal DNA amplification.

### 2.4 Carbon and nitrogen measurements

Carbon and nitrogen content was determined in leaves and roots (1 mg) by dry combustion with a Perkin Elmer Elemental Analyzer (EA 2400 Series II CHNS/O Analyzer) and expressed as the % of element in dried leaf and root material.

### 2.5 Amino acid and protein measurements

Amino acid concentration in above-ground biomass was determined by HPLC-DAD for each treatment. 100 mg of leaf material was homogenized and used for amino acid extraction as described in González-Teuber et al. (2023). Protein concentration in leaves and roots was measured using Bradford’s reagent (Bradford, 1976), with bovine serum albumin used as a standard.

### 2.6 Carbohydrate measurements

100 mg of leaf and root material was homogenized and extracted with methanol/chloroform/water (12:5:3 v/v/v). Supernatant was used for analysis of total soluble sugars (TSS) and remaining residues were kept at -20°C for starch determination. TSS were determined using 2% phenol and sulfuric acid (Dickson, 1979; Chow and Landhäusser, 2004). Starch was hydrolyzed to glucose using a sodium acetate buffer and amyloglucosidase (Sigma-Aldrich 10115, St. Louis, MO, USA) at 45°C and measured with a phenol-sulfuric acid reaction (Marquis et al., 1997). Both TSS and starch concentrations were determined spectrophotometrically at 490 nm with an Infinite 200 PRO (Tecan) using sucrose and glucose, respectively, as standards. Non-structural carbohydrates (NSC) were calculated by adding TSS and starch concentrations.

### 2.7 Measurements of Glutamine Synthetase (GS) and Glutamate Dehydrogenase (GDH) activities

To explore mechanisms of nitrogen assimilation in leaves, GS and GDH have been measured. Both are key enzymes in plant nitrogen metabolism, responding adaptively to low nitrogen availability in diverse crop species, including *C quinoa* (Bascuñán-Godoy et al., 2018). The primary pathway is constituted by GS enzyme, and alternate pathway followed by GDH enzyme (Miflin and Habash, 2002; Song et al., 2022). GS activity (EC 6.3.1.2) was measured by the formation of γ-glutamyl hydroxamate using the transferase assay (Lea et al., 1990). 100 mg of fresh Quinoa leaves were ground into a powder in an ice-chilled mortar with liquid N_2_ and suspended in a 500 µL of homogenization buffer (100 mM Tris-HCl buffer, pH 7.8, containing 3.3 mM MgCl_2_, 10 mM β-mercaptoethanol, 1 mM dithiothreitol, 15% v/v ethylene glycol). The mixture for the GS essay contained 500 μL of reaction buffer (80 mM glutamic acid, 20 mm MgSO_4_, 8 mM ATP, 6 mM hydroxylamine, 1 mM ethylenediaminetetraacetic acid, 0.1 mM Tricine, pH 7.8). The reaction was initiated by the addition of 200 µL of enzyme extract, incubated at 30°C per 15 min, and then terminated by the addition of 700 µL of ferric chloride reagent (0.67 mM FeCl_3_, 0.37 M HCl and 20% v/v trichloroacetic acid). Finally, the optical density of the supernatant was determined spectrophotometrically at 540 nm.

Glutamate dehydrogenase (GDH) activity (EC 1.4.1.4) was determined according to the procedure outlined by Kumar et al. (2000). Leaf enzyme extract was used for the determination of GDH. The mixture for the GDH-NADH assay contained 1400 µL of reaction buffer (100 mM Tris-HCl, 20 mM ketoglutarate, 150 mM (NH_4_)_2_SO_4_, 0.2 mM NADH and 1 mM MgCl_2_) or GDH-NAD^+^ essay contains 1400 µL of reaction buffer (100 mM Tris-HCl, 50 mM L-glutamate, 0.6 mM NAD^+^). The reaction was initiated by the addition of 100 µL of enzyme extract and absorbance determined spectrophotometrically at 340 nm. GDH activity was expressed as one unit of enzyme activity in terms of the amount of enzyme required to oxidize or reduce 1 nmol of NADH or NAD^+^ min^-1^ mg^-1^ protein.

### 2.8 Plant photosynthetic and morphological parameters

Gas exchange measurements of net photosynthesis (A_N_) (μmol CO_2_ m^-2^ s^-1^), stomatal conductance (gs) (mmol H_2_O m^-2^ s^-1^), and transpiration (T) (mmol H_2_O m^-2^ s^-1^) were performed for fully expanded leaves (third leaf from the top) using a portable open gas exchange system (CIRAS-2, PP Systems Amesbury, MA, USA). A_N_, g_s_, and T rates were measured at mid-morning (between 9 a.m. and 2 p.m.) after gas exchange had stabilized. Conditions in the leaf chamber were as follows: temperature at 25°C, 50% relative humidity, CO_2_ concentration 400 mol mol^-1^ and 1,000 μmol photon m^-2^ s^-1^. Leaves were first equilibrated for at least 5 min in 400 μmol mol^-1^ of external CO_2_ in a leaf cuvette. At the end of the experiment, half of the plants of each treatment were divided into above- (shoots) and below-ground (roots) tissues for weighing. Roots were first washed with tap water, and then roots and shoots were oven-dried separately at 60°C for 72 h. Fresh and dry weights of each were derived pre and post drying, respectively.

### 2.9 Fungal DNA amplification in roots

*Beauveria* and *Metarhizium* DNA was extracted from fresh mycelium pure culture and roots of three plants (50 - 200 mg of fresh tissue) using NucleoSpin^®^ Plant II MACHEREY-NAGEL Kit according to the manufacturer’s protocol. The extracted DNA was stored at -20°C for subsequent detection and specific primer amplification analyses. Primer for *Beauveria* spp. and *Metarhizium* spp. detections are listed in **Supplementary Material Table S1**. qPCR analysis was performed on an Agilent Mx3000P QPCR system (Agilent Technologies, USA) using Brilliant II SYBR Green qPCR Master Mix (Agilent Technologies, USA). Each qPCR reaction contained 7.5 of II SYBR Green qPCR Master Mix (Agilent Technologies, USA), 5 µl of gDNA, and 10 ng/µl of each primer in a final volume of 15 µL. The thermocycling program was set as: *Beauveria* spp., 95°C for 10 min, followed by 40 cycles of 95°C for 30 s, 54°C for 30s, 72°C for 40 s, and *Metarhizium* spp., 95°C for 10 min, followed by 40 cycles of 90°C for 15 s, 60°C for 15 s, and 72°C for 25 s. qPCR amplification were performed in triplicate for each template dilution. The threshold line and the sample specific threshold cycle numbers (C_T_) were determined with the default parameters of the software Agilent Aria Real-Time PCR system (Agilent Technologies, USA). Standard quantification curves consisted of the C_T_ diluted values plotted against the logarithm of the number of gDNA amount that were calculated for each standard quantification curve from *Beauveria* spp. or *Metarhizium* spp. pure culture between 2.5 to 2,500 pg and relate C_T_ values according to Smith and Osborn (2009). The validation analysis was performed with three independent biological replicates. The specificity of each primer pair was verified by determining the melting curves at the end of each run. The quality of primers product was confirmed by gel electrophoresis (**Supplementary Figure S1**).

### 2.10 Statistical analysis

Before any statistical analysis, the data were transformed as necessary to achieve normality and homogeneity of residuals. Considering the high dispersion in the data, outliers were discarded using the criteria of the Rosner (Rosner, 1975) and Dixon tests (Barnett and Lewis, 1995). A two-way analysis of variance (ANOVA) was conducted to assess the effects of the presence/absence of EIPF and N level on morphological, physiological, and biochemical responses in *C. quinoa*. A *post hoc* Fisher’s LSD test was performed to analyze differences among treatments. All analyses were conducted in R Studio (R Core Team, 2024).

## 3 Results

### 3.1 Effects of N level and EIPF on N content, proteins and amino acids

**Figure 1:**
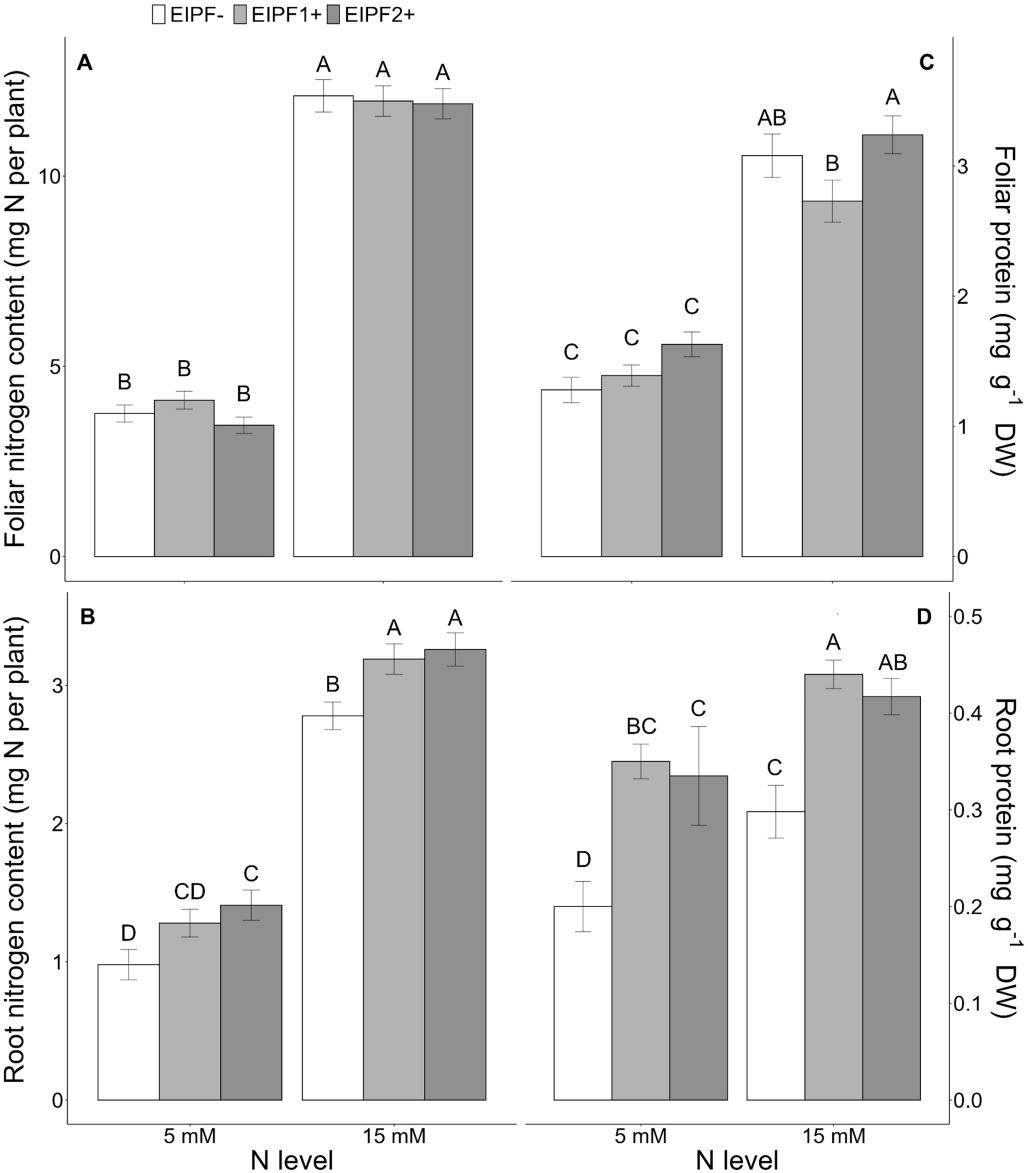
Effects of nitrogen (N) level and EIPF inoculation on foliar and root N and protein contents in *C. quinoa*. **(A)** foliar N content (n = 8-9), **(B)** root N content (n = 6-8), **(C)** foliar protein content (n = 7-8), and **(D)** root protein content (n = 5-6). Error bar labels with different letters indicate significant differences (P < 0.05) among treatments. 5 mM, low nitrogen level; 15 mM, high nitrogen level; EIPF-, non-inoculated plants; EIPF1+, inoculated with *Beauveria*; EIPF2+, inoculated with *Metarhizium*.

While foliar N content was significantly affected by N level, but not by EIPF (**Table 1**; **Figure 1A**), root N content was significantly affected by both N level and EIPF (**Table 1**). At both N levels root N content was significantly higher in EIPF-treated plants than in EIPF- plants (**Figure 1B**). For proteins, both foliar and root protein concentrations were significantly affected by N level as well as by EIPF inoculation (**Table 1**). For foliar proteins, under both N levels no positive effects of EIPF were observed relative to EIPF- plants (**Figure 1C**). In contrast, root proteins under both N levels were considerably higher in EIPF-inoculated plants (an increase higher than 30% for both EIPF1+ and EIPF2+ plant groups) compared to EIPF- plants (**Figure 1D**). Foliar total and single amino acid concentrations were significantly affected by N level; nevertheless, no significant effects of EIPF were observed on them (**Supplementary Table S2**). Not surprisingly, amino acids increased under high N levels compared to low N conditions (**Supplementary Table S2**).

**Table 1:** Two-way ANOVA of the effects of nitrogen (N) level and EIPF inoculation on physiological and morphological traits in *Chenopodium quinoa*.

	F-*value*			Replicates
	N	EIPF	N × EIPF	
Foliar N (mg N per plant)	**1012.70** **	1.12NS	0.89NS	8-9
Root N(mg N per plant)	**382.61** **	**8.88** **	0.12 NS	6-8
Foliar proteins(mg proteins g^-1^ dry weight)	**223.12** **	**4.50** **	1.56NS	7-8
Root proteins(mg proteins g^-1^ dry weight)	**16.63** **	**15.71** **	0.02NS	5-6
Foliar C (mg C per plant)	**723.39** **	**6.67** **	2.43NS	8-9
Root C(mg C per plant)	**154.61** **	4.02*	0.56NS	6-8
Foliar NSC (mg g^-1^ dry weight)	0.96NS	**7.49** **	1.24NS	5-9
Root NSC(mg g^-1^ dry weight)	**21.62** **	0.35NS	**3.95** **	5-10
GS(nmol Glu min^-1^ mg^-1^ proteins)	**5.81** *	**8.49** **	**7.37** **	4-6
GDH-NADH(nmol NADH min^-1^ mg^-1^ proteins)	**13.55** **	2.02NS	0.13NS	4
GDH-NAD^+^(nmol NAD^+^ min^-1^ mg^-1^ proteins)	**23.02** **	0.28NS	0.06NS	4
Net photosynthesis (µmol CO_2_ m^-2^ s^-1^)	**49.02** **	**5.59** **	2.49NS	4-6
Stomatal conductance (mmol H_2_O m^-2^ s^-1^)	**11.36** **	**17.73** **	1.48NS	4-6
Transpiration (mmol H_2_O m^-2^ s^-1^)	**7.38** *	**18.57** **	2.24NS	4-6
Above-ground biomass (g dry weight)	**508.23** **	**9.43** **	1.51NS	11-15
Below-ground biomass (g dry weight)	**184.37** **	2.34NS	0.23NS	11-15
Total biomass (g dry weight)	**449.65** ***	**6.48** **	1.06NS	11-15

Nitrogen (N) level - LN, low nitrogen: 5 mM and HN, high nitrogen: 15 mM. EIPF-, non-inoculated plants; EIPF1+, inoculated with *Beauveria*; EIPF2+, inoculated with *Metarhizium*). F values are shown; * indicates significance at the 0.05 level, ** indicates significance at the 0.01 level, whereas *** indicates significance at the 0.001 level. NS indicates no significant difference. Bold values denote statistical significance at the p < 0.05 level.

### 3.2 Effects of N and EIPF on carbon content and carbohydrates

**Figure 2:**
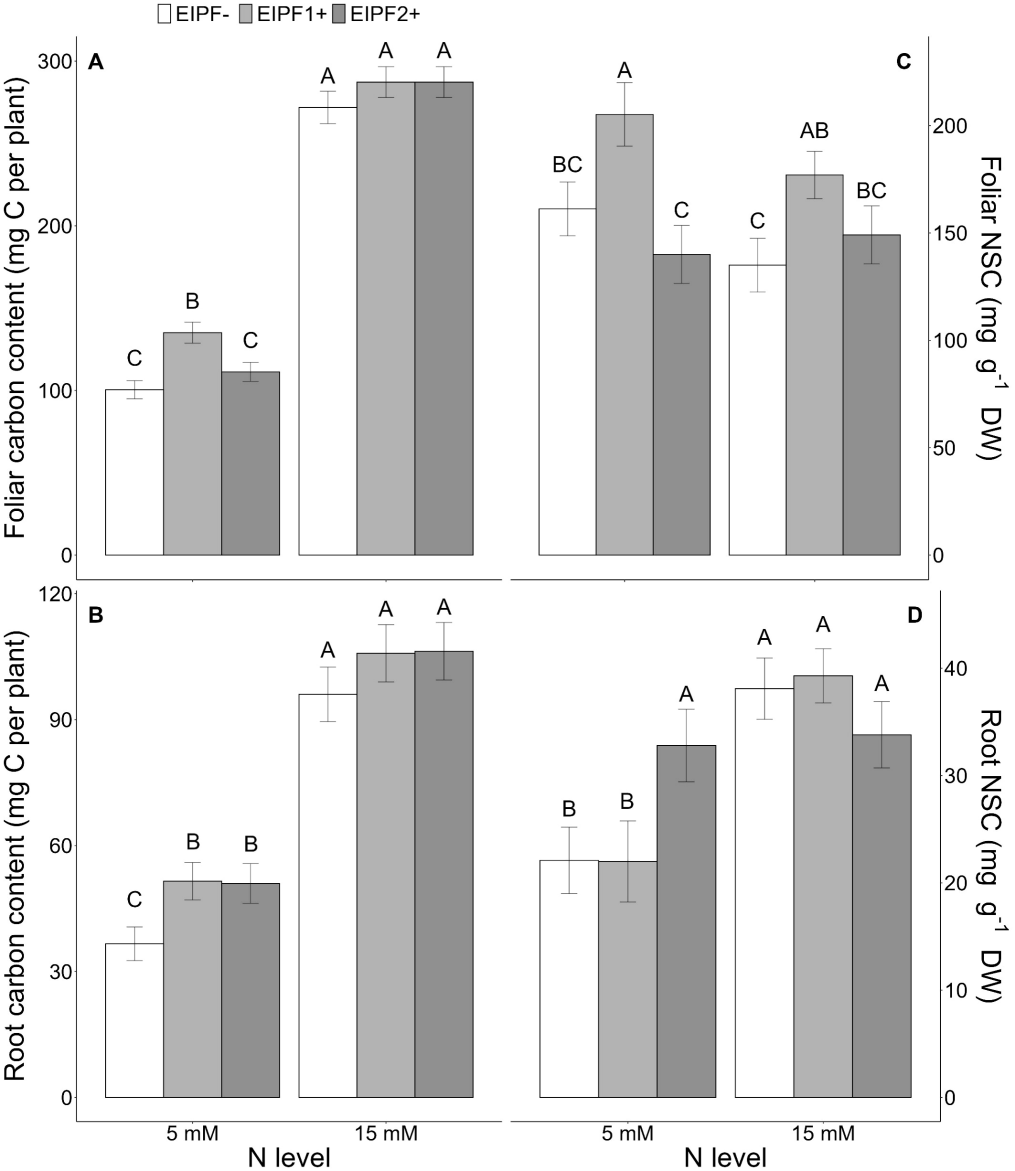
Effects of nitrogen (N) level and EIPF inoculation on foliar and root C and non-structural carbon (NSC) contents in *C. quinoa*. **(A)** foliar carbon content (n = 8-9), **(B)** root carbon content (n = 6-8), **(C)** foliar NSC content (n = 5-9), and **(D)** root NSC content (n = 5-10). Error bar labels with different letters indicate significant differences (P < 0.05) among treatments. 5 mM, low nitrogen level; 15 mM, high nitrogen level; EIPF-, non-inoculated plants; EIPF1+, inoculated with *Beauveria*; EIPF2+, inoculated with *Metarhizium*.

Both foliar and root C content were significantly affected by N level and EIPF inoculation (**Table 1**). Foliar C content was only improved under low N levels by EIPF1+; at high N levels no effects by EIPF were observed on this trait (**Figure 2A**). For root C content, positive effects of both EIPF were only observed under low N levels; no effects were evident under high N conditions (**Figure 2B**). Whereas leaf NSC was significantly affected by EIPF1+ under both N levels, no effects of EIPF2+ was observed on this trait regardless of N level (**Figure 2C**). Contrary, root NSC was only significantly affected by EIPF2+ under low N levels; no effects of EIPF were evident under high N levels (**Figure 2D**). Effects of N level and EIPF on TSS and starch concentration are shown in **Table 2**. Under low N levels no significant differences in foliar TSS were observed among EIPF+ and EIPF- plants; nevertheless, under high N levels only EIPF1+ positively affected foliar TSS in plants (**Table 2**). For root TSS, under low N levels, there was a tendency that both EIPF improved this trait in *C. quinoa* plants relative to EIPF-; nevertheless, significant differences were observed only for EIPF2+. In contrast, under high N levels no significant differences in root TSS were observed between EIPF- and EIPF+ plants (**Table 2**). For foliar starch, under low N levels, no significant differences were observed between EIPF- and EIPF+ plants; nevertheless, under low N levels foliar starch was considerably improved by EIPF1+ colonization (**Table 2**). For root starch, no significant effects by EIPF were observed neither under low nor under high N levels (**Table 2**).

**Table 2:** Effects of nitrogen (N) level and EIPF inoculation on foliar and root total soluble sugars and starch in *Chenopodium quinoa*.

	EIPF-		EIPF1+		EIPF2+		F-*value*		
	LN	HN	LN	HN	LN	HN	N	EIPF	N × EIPF
Foliar TSS (mg g^-1^ DW)	6.2 ± (0.40) CD	7.18 ± (0.46) BC	5.85 ± (0.49) CD	9.46 ± (0.47) A	5.8 ± (0.45) D	7.98 ± (0.53) B	35.89 **	2.64 NS	**3.49** *
Root TSS (mg g^-1^ DW)	6.7 ± (0.70) C	11.0 ± (0.65) A	8.47 ± (0.77) BC	10.5 ± (0.54) A	9.51 ± (0.70) AB	10.3 ± (0.65) AB	19.45 **	0.89 NS	**3.46** *
Foliar starch (mg g^-1^ DW)	167 ± (11) AB	134 ± (10.1) C	177 ± (12) A	170 ± (8.21) A	139 ± (11) BC	138 ± (10.1) BC	1.64 NS	**6.14** **	1.31 NS
Root starch (mg g^-1^ DW)	17.8 ± (2.91) BC	28.9 ± (2.44) A	12.5 ± (3.45) C	26.6 ± (2.72) A	24.2 ± (2.91) AB	23.8 ± (3.15) AB	11.95 **	0.97 NS	**3.33** *

Total soluble sugars (TSS) (n = 5–9); starch (n = 5–10). Data represent means ± (standard error). Different letters represent significant differences between N levels (LN, low nitrogen: 5 mM and HN, high nitrogen: 15 mM) and EIPF (without inoculation EIPF-; with *Beauveria*, EIPF1+; with *Metarhizium*, EIPF2+). F values are shown; * indicates significance at the 0.05 level, ** indicates significance at the 0.01 level, whereas *** indicates significance at the 0.001 level. NS indicates no significant difference. Bold values denote statistical significance at the p < 0.05 level.

### 3.3 Effects of N level and EIPF on enzyme activities

**Figure 3:**
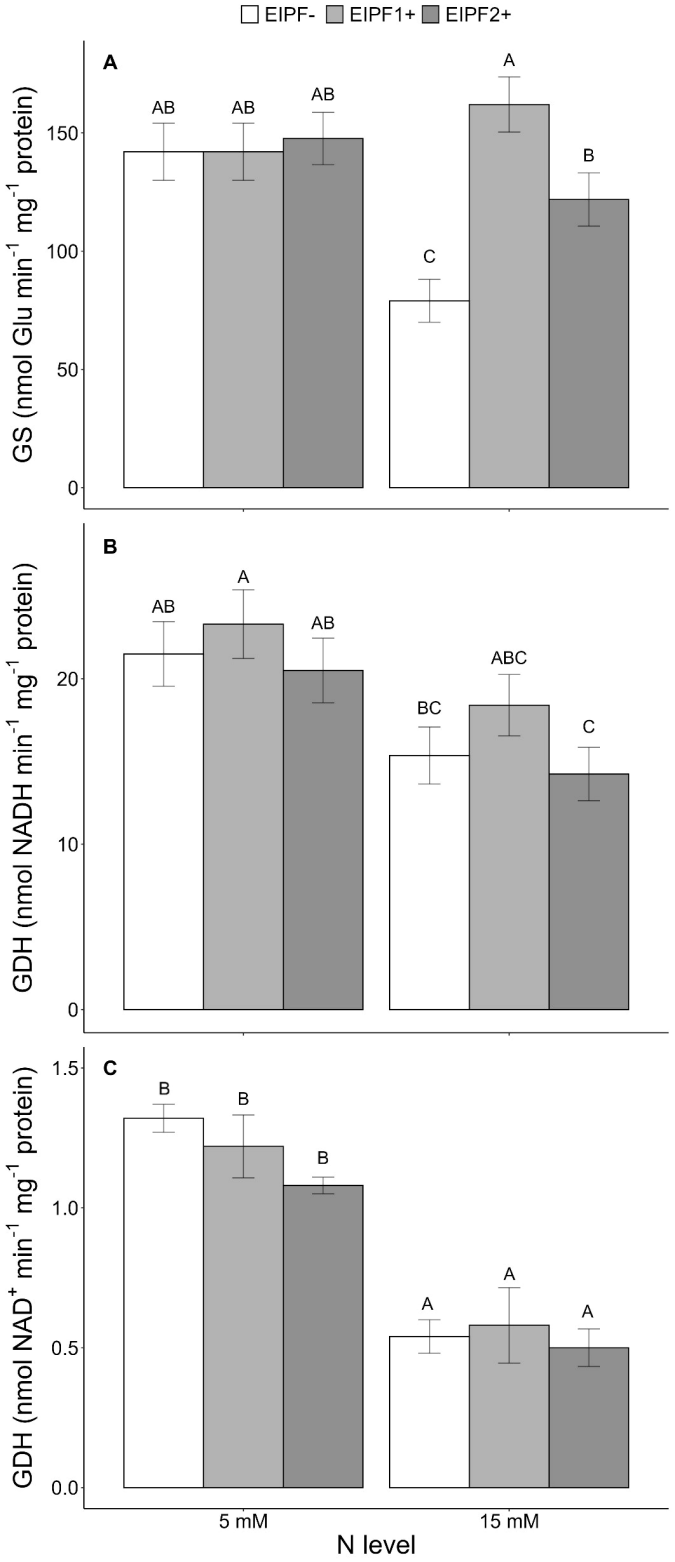
Effects of nitrogen (N) level and EIPF inoculation on foliar enzyme activities in *C. quinoa*. **(A)** glutamine synthetase (GS) (n = 4-6), **(B)** glutamate dehydrogenase (GDH-NADH) (n = 4), and **(C)** glutamate dehydrogenase (GDH-NAD+) (n = 4). Error bar labels with different letters indicate significant differences (P < 0.05) among treatments. 5 mM, low nitrogen level; 15 mM, high nitrogen level; EIPF-, non-inoculated plants; EIPF1+, inoculated with *Beauveria*; EIPF2+, inoculated with *Metarhizium*.

There was a significant effect of N level and EIPF inoculation on GS activity (**Table 1**). Under low N levels no differences were observed in GS activity inoculated and non-inoculated plants (**Figure 3A**). In contrast, under high N levels GS activity was considerably improved by EIPF1+ and EIPF2+ inoculation (**Figure 3A**). For GDH-NADH and GDH-NAD^+^ activities only a significant effect of N level was observed (**Table 1**). Both activities were higher under low N levels than under high N levels (**Figures 3B, C**). Nevertheless, for both enzymes no differences were detected between inoculated and non-inoculated plants regardless of N level (**Figures 3B, C**).

### 3.4 Effects of N level and EIPF on photosynthetic traits

**Figure 4:**
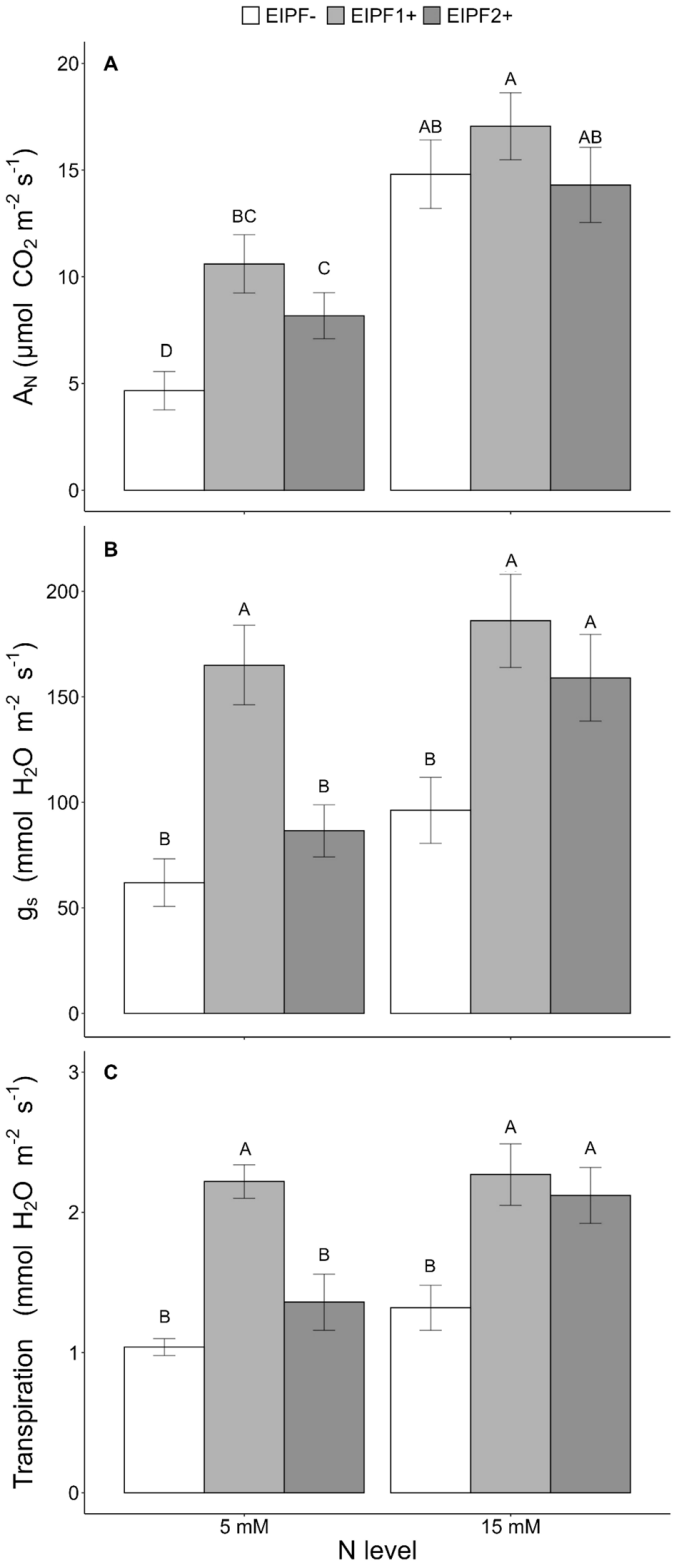
Effects of nitrogen (N) level and EIPF inoculation on photosynthetic parameters in *C. quinoa* **(A)** net photosynthetic rate (µmol CO_2_ m^−2^ s^−1^) (n = 4-6), **(B)** stomatal conductance rate (mmol H_2_O m^−2^ s^−1^) (n = 4-6), and **(C)** transpiration (mmol H_2_O m^−2^ s^−1^) (n = 4-6). Error bar labels with different letters indicate significant differences (P < 0.05) among treatments. 5 mM, low nitrogen level; 15 mM, high nitrogen level; EIPF-, non-inoculated plants; EIPF1+, inoculated with Beauveria; EIPF2+, inoculated with *Metarhizium*.

Net photosynthesis (A_N_), stomatal conductance (g_s_), and transpiration were significantly affected by N level and EIPFs (**Table 1**). Photosynthesis, but neither stomatal conductance nor transpiration, increased significantly at high N level. At low N levels, photosynthesis increased 127% and 75% by EIPF1+ and EIPF2+, respectively, relative to EIPF- (**Figure 4A**). No changes in photosynthesis were observed in response to EIPF inoculation at high N levels (**Figure 4A**). For stomatal conductance and transpiration, at low N levels only EIPF1+ triggered an increase in both traits. At high N levels both EIPF1+ and EIPF2+ increased stomatal conductance and transpiration (**Figures 4B, C**).

### 3.5 Effects of N levels and EIPF on plant growth

**Figure 5:**
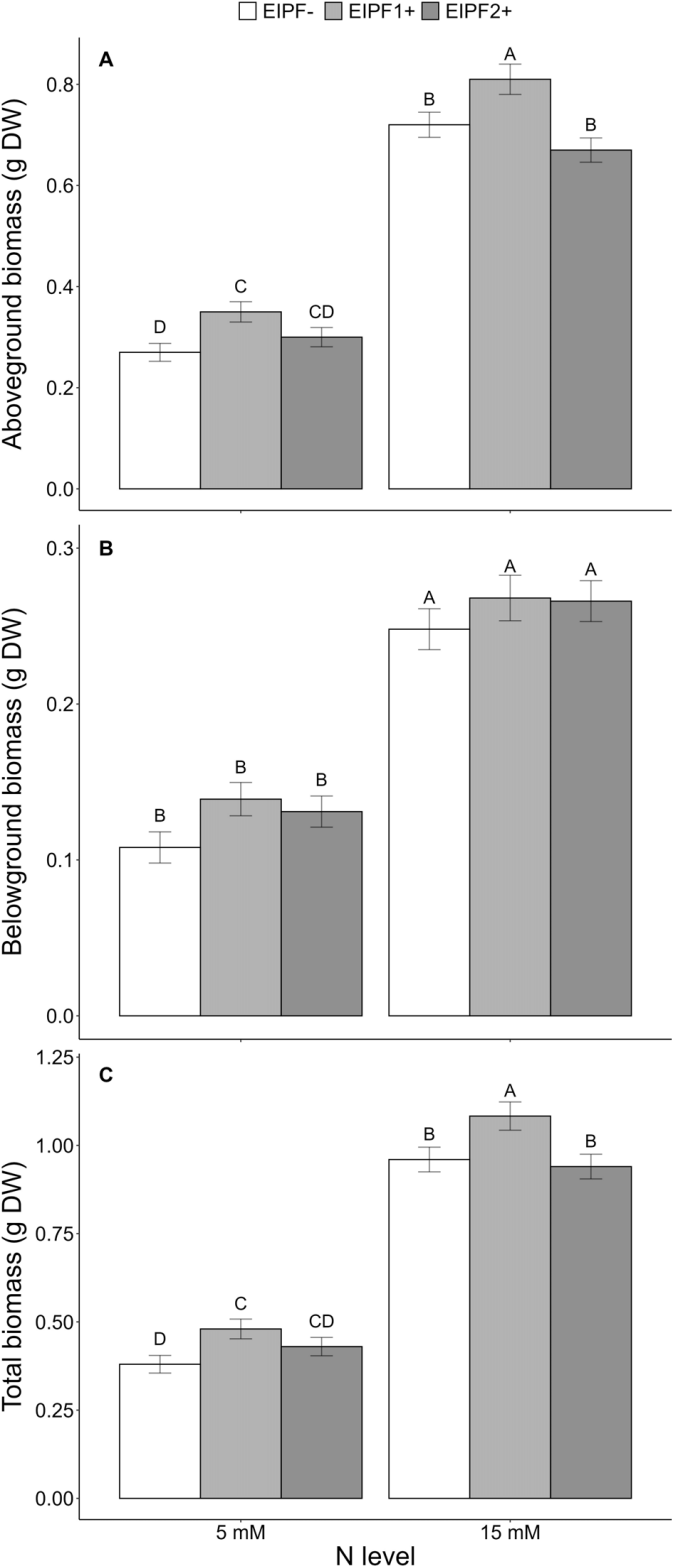
Effects of nitrogen (N) level and EIPF on plant growth (g dry weight) in *C. quinoa*. **(A)** above-ground biomass (n = 11-15), **(B)** below-ground biomass (n = 11-15), **(C)** total biomass (n = 11-15). Error bar labels with different letters indicate significant differences (P < 0.05) among treatments. 5 mM, low nitrogen level; 15 mM, high nitrogen level; EIPF-, non-inoculated plants; EIPF1+, inoculated with *Beauveria*; EIPF2+, inoculated with *Metarhizium*.

Above-ground and total biomass were significantly affected by N level and EIPF, whereas below-ground biomass was only affected by N level (**Table 1**). Plants under low N levels displayed 50% lower above-ground, below-ground and total biomass relative to high N level plants (**Figures 5A–C**). EIPF1+ significantly increased above-ground and total biomass at both N levels (**Figures 5A, C**). In contrast, EIPF2+ did not affect any biomass trait regardless of N level (**Figure 5A**).

### 3.6 Fungal DNA amplification in roots

Primers designed for each fungus were specific, generating only one PCR product whose size was consistent with the observed weight (465 kb for *Beauveria* and 337 kb for *Metarhizium*) (**Supplementary Figure S1**). Based on qPCR analysis, presence of *Beauveria* and *Metarhizium* DNA in roots was confirmed in all inoculated plants, except for uninoculated plants (EIPF-) (**Supplementary Figure S2A**). Fungal DNA obtained from *Beauveria* roots (EIPF1+) tended to be higher than in control roots at both N levels (LN, control vs *Beauveria*: F = 6.09, P = 0.069; HN, control vs *Beauveria*: F = 6.22, P = 0.061). Similarly, fungal DNA from *Metarhizium* roots (EIPF2+) was significantly higher than in control roots at both N levels (LN, control vs *Metarhizium*: F = 79.86, P = < 0.0001; HN, control vs *Beauveria*: F = 25.71, P = 0.007) (**Supplementary Figure S2B**).

## 4 Discussion

We showed that EIPF strains *Beauveria* and *Metarhizium* isolated from southern Chilean vineyards were able to translocate N from soil to roots of *C. quinoa*, with positive effects on N and C storage, photosynthesis, and plant growth. Our results are consistent with previous studies demonstrating that EIPF are able to transfer insect-derived N from soils to plants (Behie et al., 2012; Behie and Bidochka, 2014a, b; Barelli et al., 2019). Here, we showed evidence that this translocation phenomenon is also possible in the absence of soil insects.

Enhanced root N and protein content, triggered by EIPF, was observed in *C. quinoa* at both low and high N levels. Barelli et al. (2019) showed that insect-derived N transfer by the strain *Metarhizium robertsii* to *Phaseolus vulgaris* was only evident under nutrient-poor soil conditions (i.e., low carbon and nitrogen content), suggesting that nutrient supply from the host plant to the fungus is essential for maintaining the symbiosis. A similar situation occurs in plant-mycorrhizal interactions (González-González et al., 2020; Wang et al., 2020b). For example, Fellbaum et al. (2012) showed that C flux from the root to the fungus triggers the uptake and transport of N in symbiosis. Moreover, N transport is stimulated only when C is delivered by the host across the mycorrhizal interface, not when C is supplied directly to the fungal extraradical mycelium (Fellbaum et al., 2012). In our system, an improvement in root C as well as carbohydrate content was observed in the presence of EIPF, which suggests greater leaf to root translocation. This was particularly evident at low N levels, suggesting nutrient exchange between *C. quinoa* and both *Beauveria* and *Metarhizium*. Mechanisms involved in plant to fungus and fungus to plant C and N translocation have yet to be investigated for EIPF. Regarding N metabolism, our results indicate that GS and GDH increase at high foliar N levels, but not at low N levels. The foliar N recycling level is likely not high enough to induce an activation of the enzyme related with amination at LN. In contrast to that observed in roots, no differences in foliar N content between EIPF- and EIPF+ plants were observed. We cannot rule out the possibility that enhanced root N content in inoculated plants was likely incorporated into roots in other forms, such amino acids or other organic molecules. The latter is consistent with our observations of improved root protein content (Yang et al., 2020; Guo et al., 2021). In symbiosis, the role of root enzymes and N and sugar transporters are key in the exchange of these nutrients (Doidy et al., 2012; Fellbaum et al., 2012; Sun et al., 2020). Still little is known about plant carbohydrates obtained by EIPF and how they are transported into the fungus, however (Fang and St. Leger, 2010; Barelli et al., 2019). Further research is needed to understand plant-EIPF chemical communication during the establishment of the symbiosis.

Improved N transfer triggered by fungi usually relates to improved plant growth in either above- or below-ground biomass (Zhou et al., 2018; Barelli et al., 2019). This phenomenon seems to be context dependent, however, and may rely on a range of factors, such as soil nutrient availability. For example, growth benefits promoted by *Beauveria bassiana* in maize plants were only evident under high soil nutrient availability (NPK fertilizer) (Tall and Meyling, 2018). In contrast, Zhou et al. (2018) found that low N-fertilizer application promoted growth in rice and *Arabidopsis*, triggered by the fungal endophyte *Phomopsis liquidambaris*. Here, we showed that benefits on plant growth were only evident in presence of EIPF1+ (*Beauveria* strain) regardless the N level. Contrary, EIPF2+ (*Metarhizium* strain) showed positive effects in terms of photosynthesis under low N levels; nevertheless, these effects were not reflected in better plant growth. Enhanced plant biomass triggered by EIPF1+ was, however, not related to improved N transfer from below-ground to above-ground biomass, suggesting that other mechanisms are likely involved. Stomatal conductance was positively affected by EIPF, particularly by *Beauveria*, which has been associated with increases in photosynthesis and plant growth in *C. quinoa* (Bascuñán-Godoy et al., 2018). Additionally, *Beauveria* strain used in this study is able to synthesize phytohormones *in vitro*, including auxin and gibberellin (unpublished data), which may relate to plant growth promotion (Bader et al., 2020; García-Latorre et al., 2023).

In general, *Beauveria* was more beneficial in terms of plant morpho-physiological performance than *Metarhizium*. While both fungal strains improved root N transfer, benefits in terms of foliar non-structural carbohydrates (NSC) and above-ground biomass were only evident in the presence of *Beauveria*. In contrast, greater accumulation of NSC and starch was observed in below-ground biomass in the presence of *Metarhizium*. These findings suggest that symbiosis with *Metarhizium* enhanced leaf to root C allocation in *C. quinoa*, which relates to the fact that *Metarhizium* root colonization (fungal DNA abundance) was considerably higher than *Beauveria* colonization under low N levels. Thus, *Metarhizium* plants effectively trade photosynthates for nitrogen, which is translocated to the roots, possibly for maintenance and functioning of the symbiosis. In our system, *Beauveria* is likely a better partner for *C. quinoa* than *Metarhizium*. Both strains seem to be equally effective in transferring N to roots, but *Beauveria* triggered lower C allocation to roots, exerting less demand for photosynthetically derived carbohydrates relative to *Metarhizium*. Importantly, *Beauveria*, even at low root colonization, established more beneficial interactions with *C. quinoa* in terms of photosynthetic parameters and plant growth. How the plant senses and differentially rewards different EIPF partners is still unknown. Recent studies indicate that plants under stress conditions have evolved a ‘crying-for-help’ strategy, which would enable them to recruit beneficial microbial partners mediated by changes in the root exudate composition (Rizaludin et al., 2021). The outcome of the interaction, however, is difficult to generalize; it often relies on diverse factors such as abiotic factors, host plant physiology, infection intensity and genotypes of both host plant and fungal strain (González-Teuber et al., 2021).

Our study showed that symbiotic associations between *C. quinoa* and *Metarhizium* and *Beauveria* help plants to improve N transfer, even in absence of insects, with positive effects on N and C storage, photosynthesis, and plant growth. Moreover, N availability seems to be key in regulating these benefits. A better understanding of the biochemical mechanisms and underlying molecular basis is required to explain how plant and fungal partners regulate nutrient exchange in this system. Since *Beauveria* and *Metarhizium* are ubiquitous in soil ecosystems (Behie and Bidochka, 2014b) and establish associations with a wide range of plants, these EIPF have the potential to provide a sustainable alternative to chemical fertilizers in agricultural systems. Since multiple microbial symbionts may act in tandem to increase host benefits (González-Teuber et al., 2022), future research should consider testing simultaneous effects of both EIPF *Beauveria* and *Metarhizium* on plant growth promotion and nutrient exchange.

## Data Availability

The raw data supporting the conclusions of this article will be made available by the authors, without undue reservation.
