# Corrigendum: Root symbiotic fungi improve nitrogen transfer and morpho-physiological performance in *Chenopodium quinoa*

**DOI:** 10.3389/fpls.2024.1513724

**Published:** 2024-12-17

**Authors:** Shirley Alquichire-Rojas, Elizabeth Escobar, Luisa Bascuñán-Godoy, Marcia González-Teuber

**Affiliations:** ^1^ Facultad de Ciencias, Universidad Católica de la Santísima Concepción, Concepción, Chile; ^2^ Departamento de Botánica, Facultad de Ciencias Naturales y Oceanográficas, Universidad de Concepción, Concepción, Chile; ^3^ Facultad de Ciencias Biológicas, Pontificia Universidad Católica de Chile, Santiago, Chile

**Keywords:** entomopathogenic fungi, nitrogen transfer, photosynthesis, carbon allocation, plant growth, symbiosis, quinoa

In the published article, there was an error in the unit used for stomatal conductance, we wrote “g_s_ (nmol H_2_O m^-2^ s^-1^)” where we meant g_s_ (mmol H_2_O m^-2^ s^-1^). This error occurred in the Y axis title for **Figure 4B**, the caption for **Figure 4**, **Table 1** caption and in a sentence of the **Materials and methods**, section *2.8 Plant photosynthetic and morphological parameters* as published. This sentence previously stated:

“Gas exchange measurements of net photosynthesis (A_N_) (µmol CO_2_ m^-2^ s^-1^), stomatal conductance (g_s_) (nmol H_2_O m^-2^ s^-1^), and transpiration (T) (mmol H_2_O m^-2^ s^-1^) were performed for fully expanded leaves (third leaf from the top) using a portable open gas exchange system (CIRAS-2, PP Systems Amesbury, MA, USA).”

The corrected sentence appears below:

“Gas exchange measurements of net photosynthesis (A_N_) (µmol CO_2_ m^-2^ s^-1^), stomatal conductance (g_s_) (mmol H_2_O m^-2^ s^-1^), and transpiration (T) (mmol H_2_O m^-2^ s^-1^) were performed for fully expanded leaves (third leaf from the top) using a portable open gas exchange system (CIRAS-2, PP Systems Amesbury, MA, USA).”

The corrected **Figure 4** and **Table 1** are below:

**Figure 4:**
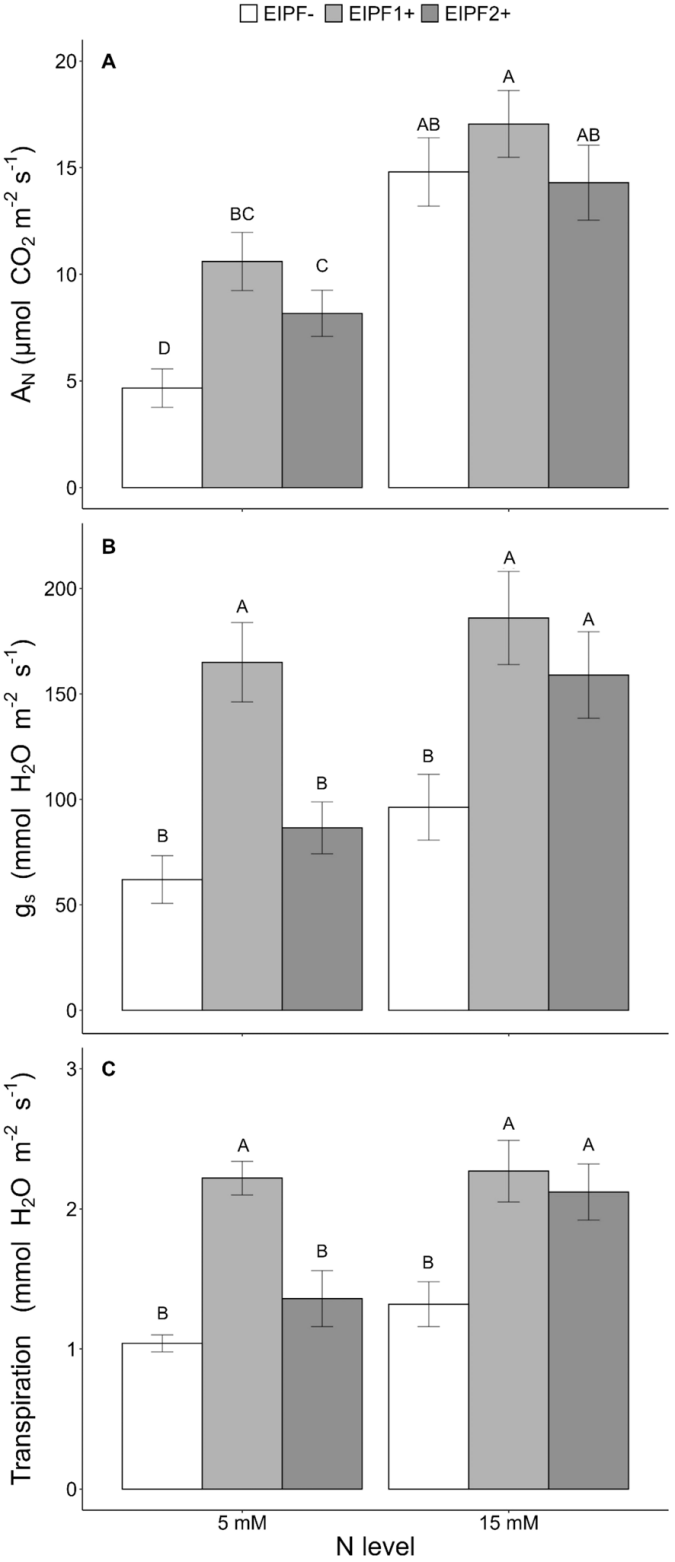
Effects of nitrogen (N) level and EIPF inoculation on photosynthetic parameters in *C. quinoa* ***(*A*)*** net photosynthetic rate (μmol CO_2_ m^-2^ s^-1^) (n = 4-6), **(B)** stomatal conductance rate (mmol H_2_O m^-2^ s^-1^) (n = 4-6), and **(C)** transpiration rate (mmol H_2_O m^-2^ s^-1^) (n = 4-6). Error bar labels with different letters indicate significant differences (P < 0.05) among treatments. 5 mM, low nitrogen level; 15 mM, high nitrogen level; EIPF-, non-inoculated plants; EIPF1+, inoculated with *Beauveria*; EIPF2+, inoculated with *Metarhizium*.

**Table 1:** Two-way ANOVA of the effects of nitrogen (N) level and EIPF inoculation on physiological and morphological traits in *Chenopodium quinoa*.

	F-*value*			Replicates
	N	EIPF	N × EIPF	
Foliar N (mg N per plant)	**1012.70** **	1.12 NS	0.89 NS	8-9
Root N (mg N per plant)	**382.61** **	**8.88** **	0.12 NS	6-8
Foliar proteins (mg proteins g^-1^ dry weight)	**223.12** **	**4.50** **	1.56 NS	7-8
Root proteins (mg proteins g^-1^ dry weight)	**16.63** **	**15.71** **	0.02 NS	5-6
Foliar C (mg C per plant)	**723.39** **	**6.67** **	2.43 NS	8-9
Root C (mg C per plant)	**154.61** **	**4.02** *	0.56 NS	6-8
Foliar NSC (mg g^-1^ dry weight)	0.96 NS	**7.49** **	1.24 NS	5-9
Root NSC (mg g^-1^ dry weight)	**21.62** **	0.35 NS	**3.95** **	5-10
GS (nmol Glu min^-1^ mg^-1^ proteins)	**5.81** *	**8.49** **	**7.37** **	4-6
GDH-NADH (nmol NADH min^-1^ mg^-1^ proteins)	**13.55** **	2.02 NS	0.13 NS	4
GDH-NAD^+^ (nmol NAD^+^ min^-1^ mg^-1^ proteins)	**23.02** **	0.28 NS	0.06 NS	4
Net photosynthesis (µmol CO_2_ m^-2^ s^-1^)	**49.02** **	**5.59** **	2.49 NS	4-6
Stomatal conductance (mmol H_2_O m^-2^ s^-1^)	**11.36** **	**17.73** **	1.48 NS	4-6
Transpiration (mmol H_2_O m^-2^ s^-1^)	**7.38** *	**18.57** **	2.24 NS	4-6
Above-ground biomass (g dry weight)	**508.23** **	**9.43** **	1.51 NS	11-15
Below-ground biomass (g dry weight)	**184.37** **	2.34 NS	0.23 NS	11-15
Total biomass (g dry weight)	**449.65** ***	**6.48** **	1.06 NS	11-15

Nitrogen (N) level - LN, low nitrogen: 5 mM and HN, high nitrogen: 15 mM. EIPF-, non-inoculated plants; EIPF1+, inoculated with *Beauveria*; EIPF2+, inoculated with *Metarhizium*). F values are shown; * indicates significance at the 0.05 level, ** indicates significance at the 0.01 level, whereas *** indicates significance at the 0.001 level. NS indicates no significant difference. Bold values denote statistical significance at the p < 0.05 level.

In the published article, there was also an error in the **Funding** statement; “Other funding was provided by ANID-Subdirección Capital Humano/Doctorado Nacional/2021-21210677 (SA-R)” was omitted.

The correct **Funding** statement appears below:

“The author(s) declare that financial support was received for the research, authorship, and/or publication of this article. This work was supported by grants ANID Fondecyt Regular N° 1230282 (MG-T) and ANID Fondecyt Regular N° 1211473 (LB-G). Other funding was provided by ANID-Subdirección de Capital Humano/Doctorado Nacional/2021-21210677 (SA-R)”.

In the published article, there was an error, in the reagent’s name used for Glutamine Synthetase assay; we wrote “hydroxyamide” where we meant “hydroxylamine”.

A correction has been made to **Materials and methods**, *2.7 Measurements of Glutamine Synthetase (GS) and Glutamate Dehydrogenase (GDH) activities*, paragraph 1. This sentence previously stated:

“The mixture for the GS essay contained 500 µL of reaction buffer (80 mM glutamic acid, 20 mm MgSO_4_, 8 mM ATP, 6 mM hydroxyamide, 1 mM ethylenediaminetetraacetic acid, 0.1 mM Tricine, pH 7.8).”

The corrected sentence appears below:

“The mixture for the GS essay contained 500 µL of reaction buffer (80 mM glutamic acid, 20 mm MgSO_4_, 8 mM ATP, 6 mM hydroxylamine, 1 mM ethylenediaminetetraacetic acid, 0.1 mM Tricine, pH 7.8).”

The authors apologize for these errors and state that this does not change the scientific conclusions of the article in any way. The original article has been updated.

In the published article, there was an error in the **Conflict of Interest** statement. A correction has been made to the **Conflict of Interest** statement:

“The authors declare that the research was conducted in the absence of any commercial or financial relationships that could be construed as a potential conflict of interest.

The handling editor [NFS] is currently organizing a Research Topic with the author [LBG].”

The Field Chief Editor Dr. Chun-Ming Liu has assessed the original manuscript, the correction, and the review process, and certifies the integrity of the review process.

