# Supplementary material for: Root symbiotic fungi improve nitrogen transfer and morpho-physiological performance in Chenopodium quinoa
Source: Front Plant Sci. 2024 Aug 26;15:1386234. doi: 10.3389/fpls.2024.1386234 (PMC11409918; doi:10.3389/fpls.2024.1386234)
Supplement: Supplementary file 1 [file DataSheet1.pdf]

Supplementary Material

Root symbiotic fungi improve nitrogen transfer and morpho-physiological performance in *Chenopodium quinoa*

Alquichire-Rojas S[1], Escobar E[2], Bascuñán-Godoy L[2], González-Teuber M[3*]

[1]Facultad de Ciencias, Universidad Católica de la Santísima Concepción, Concepción, Chile.
[2]Departamento de Botánica, Facultad de Ciencias Naturales y Oceanográficas, Universidad de Concepción, Chile.
[3]Facultad de Ciencias Biológicas, Pontificia Universidad Católica de Chile, Santiago, Chile.

*** Correspondence:**
mgonzat@uc.cl

Table S1: Primers of *Beauveria* spp. and *Metarhizium* spp. used for quantitative PCR in *C. quinoa* roots.

Specie	Primer name	Primer sequence (5´-3´)	Large (pb)	Tm (°C)	CG%	Product large (pb)	Reference
Beauveria	Bb	(F) GAACCTACCTATCGTTGCTTC	21	56.17	47.6	465	Landa et al. (2013)
		(R) ATTCGAGGTCAACGTTCAG	19	55.28	47.37		
Metarhizium clade 1	Ma	(F) CCAACTCCCAACCCCTGTGAAT	22	62.49	54.55	337	Schneider et al. (2011), Ponchon et al. (2022)
		(R) AAAACCAGCCTCGCCGAT	18	55.56	55.56		

Table S2: Total and single amino acid concentrations (n = 5-6) for all plant groups are indicated. Data represent means ± (standard error). Different letters represent significant differences between EIPF (without inoculation, EIPF-; with *Beauveria*, EIPF1+; with *Metarhizium*, EIPF2+) and N levels (LN, low nitrogen and HN, high nitrogen) at a P < 0.05 level (Fisher's LSD test). Effects of N, EIPF and N x EIPF are indicated with P-values (Two-way ANOVA).

	EIPF-		EIPF1+		EIPF2+		*P*- value		
	LN	HN	LN	HN	LN	HN	N	EIPF	N × EIPF
Total amino acids	40.3 ± (3.20) B	54.0 ± (3.67) A	38.4 ± (2.83) B	56.1 ± (3.41) A	33.9 ± (2.66) B	61.6 ± (3.93) A	**< 0.0001**	0.9380	0.1860
Aspartic acid	5.33 ± (0.35) B	7.78 ± (0.39) A	5.29 ± (0.32) B	8.35 ± (0.41) A	4.97 ± (0.31) B	8.12 ± (0.44) A	**< 0.0001**	0.7150	0.6530
Glutamine + Glutamic acid	6.49 ± (0.567) B	10.0 ± (0.567) A	6.42 ± (0.518) B	10.9 ± (0.518) A	6.12 ± (0.518) B	10.3 ± (0.567) A	**< 0.0001**	0.6520	0.6710
Serine	2.56 ± (0.289) BC	3.17 ± (0.289) AB	2.44 ± (0.264) BC	3.49 ± (0.264) A	2.05 ± (0.264) C	3.85 ± (0.289) A	**< 0.0001**	0.9350	0.1230
Histidine	0.569 ± (0.051) BC	0.581 ± (0.051) BC	0.563 ± (0.047) C	0.702 ± (0.047) AB	0.453 ± (0.047) C	0.738 ± (0.051) A	**0.0009**	0.4814	**0.0394**
Glycine	2.45 ± (0.215) B	3.65 ± (0.215) A	2.37 ± (0.196) B	3.90 ± (0.196) A	2.23 ± (0.196) B	3.82 ± (0.215) A	**< 0.0001**	0.8270	0.6000
Threonine	1.90 ± (0.295) BC	2.66 ± (0.35) AB	1.98 ± (0.276) BC	2.95 ± (0.336) A	1.56 ± (0.062) C	3.34 ± (0.392) A	**0.0001**	0.8422	0.2298

Arginine	1.23 ± (0.157) BC	1.49 ± (0.157) AB	1.27 ± (0.143) BC	1.65 ± (0.143) AB	0.97± (0.143) C	1.89 ± (0.157) A	**0.0002**	0.8004	0.0797
Alanine	4.20 ± (0.271) B	5.71 ± (0.315) A	4.12 ± (0.245) B	6.55 ± (0.338) A	4.00 ± (0.015) B	6.25 ± (0.331) A	**< 0.0001**	0.5570	0.3150
Proline	0.496 ± (0.086) BC	0.654 ± (0.096) AB	0.339 ± (0.078) C	0.794 ± (0.078) A	0.445 ± (0.078) BC	0.662 ± (0.096) AB	**0.0002**	0.9676	0.1872
Tyrosine	1.26 ± (0.127) BC	1.37 ± (0.127) AB	1.21± (0.116) BC	1.504 ± (0.116) AB	1.00 ± (0.116) C	1.72 ± (0.127) A	**0.0008**	0.9413	**0.0561**
Valine	1.97 ± (0.212) B	4.25 ± (0.273) A	2.08 ± (0.193) B	3.99 ± (0.212) A	1.63 ± (0.193) B	3.94 ± (0.212) A	**< 0.0001**	0.2990	0.5660
Methionine	0.476 ± (0.062) BC	0.588 ± (0.069) AB	0.580 ± (0.062) AB	0.688 ± (0.068) A	0.396 ± (0.051) C	0.731± (0.077) A	**0.0017**	0.2551	0.1359
Isoleucine	1.54 ± (0.191) CD	1.87 ± (0.210) ABC	1.63 ± (0.179) BCD	2.16 ± (0.205) AB	1.29 ± (0.159) D	2.34 ± (0.235) A	**0.0004**	0.6640	0.2112
Leucine	3.76 ± (0.541) B	5.66 ± (0.541) A	4.10 ± (0.494) B	3.65 ± (0.494) B	3.69 ± (0.494) B	5.14± (0.541) AB	**0.0441**	0.2818	0.0662
Phenylalanine	1.92 ± (0.182) BC	2.36 ± (0.182) AB	1.99 ± (0.166) BC	2.48 ± (0.166) A	1.60 ± (0.166) C	2.72 ± (0.203) A	**0.0137**	0.4488	0.1043
Lysine	1.86 ± (0.315) B	2.31 ± (0.354) AB	1.96 ± (0.297) B	2.95 ± (0.364) A	1.53 ± (0.262) B	2.99 ± (0.402) A	**0.0011**	0.5347	0.3376

Supplementary Figure legend

Figure S1: Determination of the primer's specificity from qPCR in pure culture of EIPF. PCR amplification products of 465 bp length obtained from *Beauveria* spp. (namely B) and 337 bp obtained from *Metarhizium* spp. (namely M). DNA templates include: **(A)** genomic DNA of *Beauveria* spp., and **(D)** *Metarhizium* spp. and 1 kb marker ladder (NEB.N3232S), followed by amplification curve of primer product from qPCR of **(B)** *Beauveria* spp., and **(E)** *Metarhizium* spp., dark lines refers to tested primer product and light lines refers to control primer product (B or M), **(C)** calibration curve of *Beauveria* spp. primer product, and **(F)** calibration curve of *Metarhizium* spp. primer product.

Figure S2: Quantification of DNA of *Beauveria* and *Metarhizium* on roots of *C. quinoa* using qPCR. Boxplots represent the picograms of DNA copies per five microliters extracted of non-disinfected Quinoa roots without adhering soil (15 days post inoculation). **(A)** DNA copies in *C. quinoa* plants inoculated with EIPF1+ (*Beauveria* spp.) relative to EIPF- (non-inoculated plants), and **(B)** DNA copies in *C. quinoa* plants inoculated with EIPF2+ (*Metarhizium* spp.) relative to EIPF- (non-inoculated plants).

Figure S1

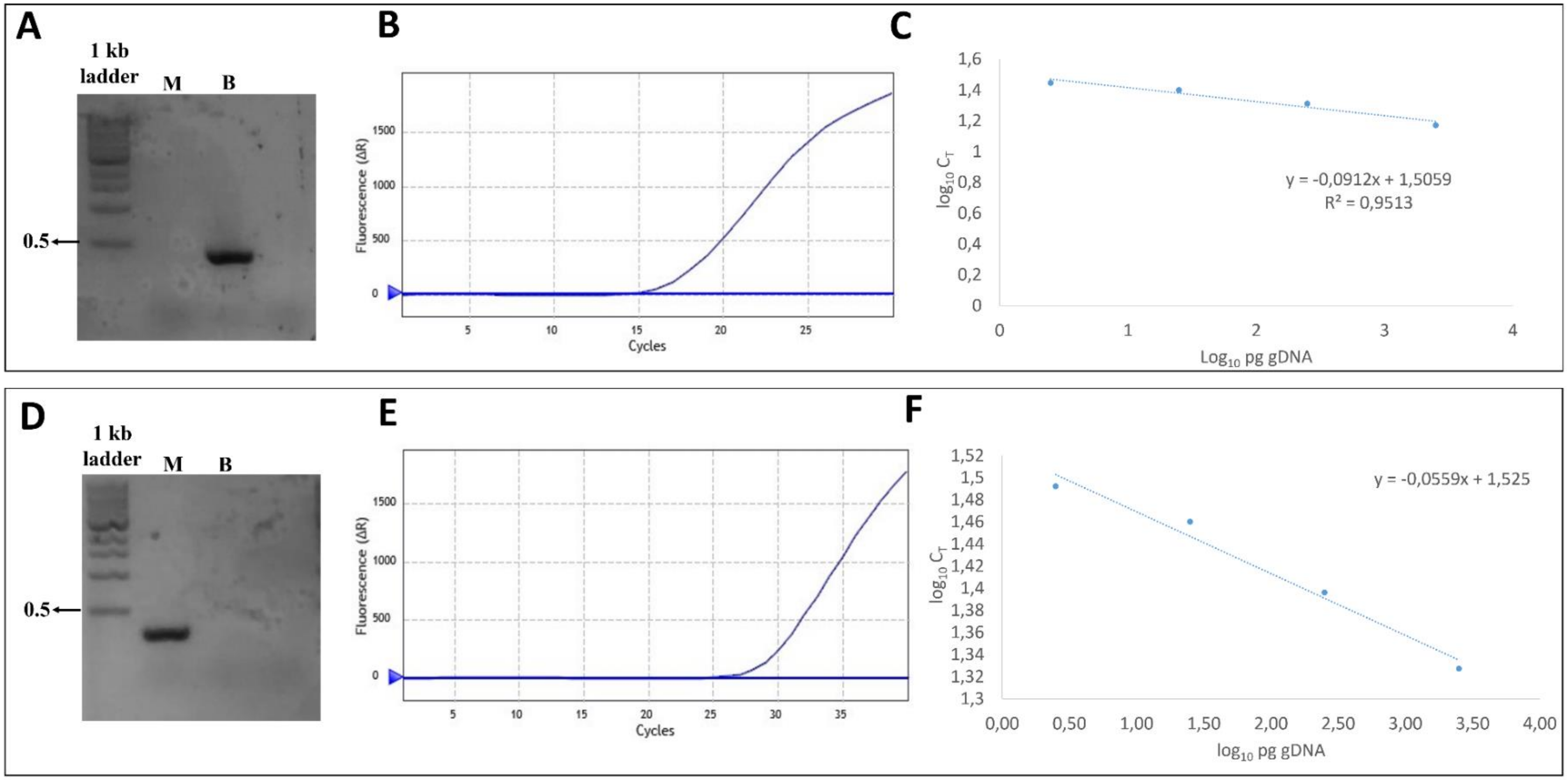
A
1 kb ladder
M
B
0.5
B
Fluorescence (ΔR)
Cycles
C
log10 CT
Log10 pg gDNA
y = -0,0912x + 1,5059
R² = 0,9513
D
1 kb ladder
M
B
0.5
E
Fluorescence (ΔR)
Cycles
F
log10 CT
log10 pg gDNA
y = -0,0559x + 1,525

Figure S2

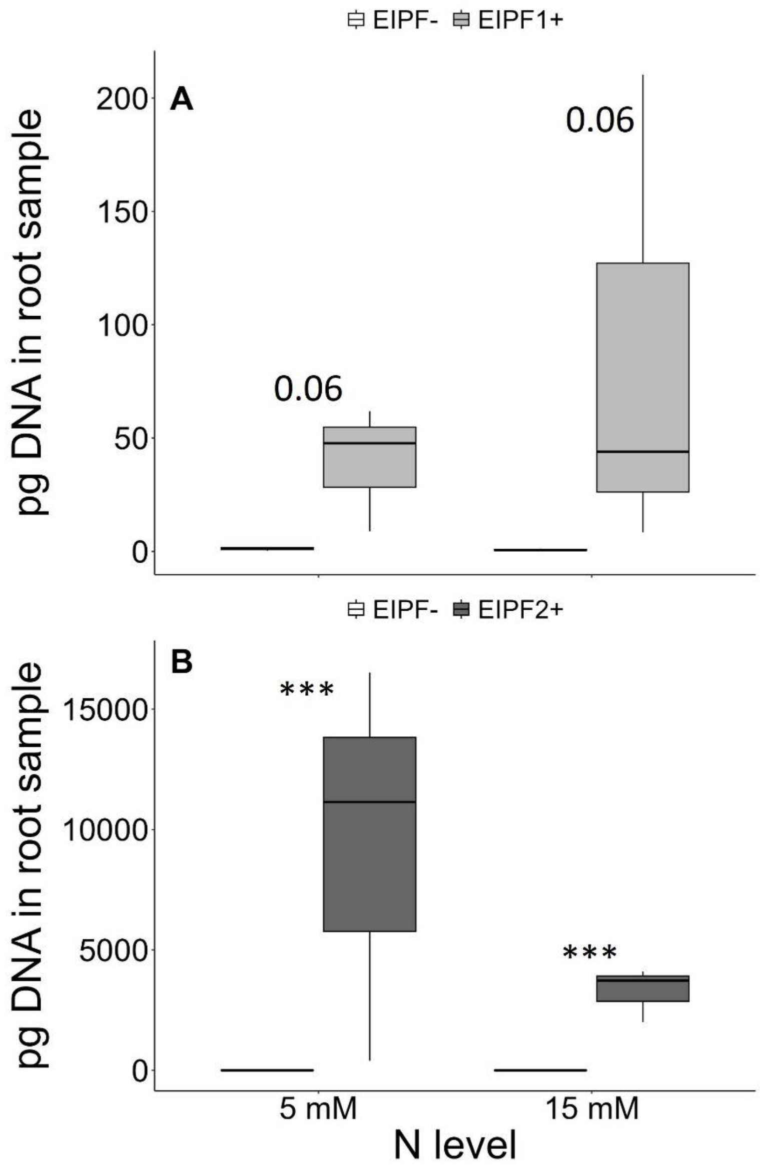
EIPF-
EIPF1+
A
pg DNA in root sample
200
150
100
50
0
0.06
0.06
EIPF-
EIPF2+
B
15000
10000
5000
0
***
***
5 mM
15 mM
N level
